# Sex effects of dietary protein source and acid load on renal and bone status in the *Pah^enu2^* mouse model of phenylketonuria

**DOI:** 10.14814/phy2.14251

**Published:** 2019-10-24

**Authors:** Bridget M. Stroup, Sangita G. Murali, Denise J. Schwahn, Emily A. Sawin, Emma M. Lankey, Hans Peter Bächinger, Denise M. Ney

**Affiliations:** ^1^ Department of Nutritional Sciences University of Wisconsin‐Madison Madison Wisconsin; ^2^ Department of Molecular and Human Genetics Baylor College of Medicine Houston Texas; ^3^ Zoetis, Inc. Kalamazoo Michigan; ^4^ Department of Biochemistry and Molecular Biology Oregon Health Sciences University Portland Oregon

**Keywords:** Bone mineral content, glycomacropeptide, kidney, renal acid load

## Abstract

The low‐phenylalanine (Phe) diet with amino acid (AA) medical foods is associated with low bone mineral density (BMD) and renal dysfunction in human phenylketonuria (PKU). Our objective was to determine if diets differing in dietary protein source and acid load alter bone and renal outcomes in *Pah^−/−^* and wild‐type (WT) mice. Female and male *Pah^−/−^* (*Pah^enu2/enu2^)* and WT littermates (C57BL/6 background) were fed high‐acid AA, buffered AA (BAA), glycomacropeptide (GMP), or high‐Phe casein diets from 3 to 24 weeks of age. The BAA diet significantly reduced the excretion of renal net acid and ammonium compared with the AA diet. Interestingly, the BAA diet did not improve renal dilation in hematoxylin and eosin (H&E) stained renal sections, femoral biomechanical parameters, or femoral bone mineral content (BMC). Significantly lower femoral BMC and strength occurred in *Pah^−/−^* versus WT mice, with greater decline in female *Pah^−/−^* mice. Polyuria and mild vacuolation in the proximal convoluted tubules were observed in male *Pah^−/−^* and WT mice fed the high‐acid AA diet versus absent/minimal cortical vacuolation in males fed the GMP, BAA, or casein diets. Vacuole contents in male mice were proteinaceous. Cortical vacuolation was absent in female mice. Dilated medullary tubules were observed in all *Pah^−/−^* mice, except for male *Pah^−/−^* mice fed the GMP diet. In summary, the PKU genotype and diet showed differential effects on renal and bone status in male and female mice. Renal status improved in male *Pah^−/−^* mice fed the GMP diet.

## Introduction

Phenylalanine hydroxylase (PAH; EC 1.14.16.1) deficiency commonly called phenylketonuria (PKU) is an autosomal recessive inborn error of Phe metabolism resulting in an inability to synthesize Tyr and elevated concentrations of its precursor Phe in blood and brain (Singh et al. 2014; Vockey et al. 2014). PKU causes severe cognitive impairment unless treated with a low‐Phe diet shortly after birth (Singh et al. 2014). The cornerstone of PKU management is life‐long adherence to a low‐Phe diet restricted in protein from all foods, except limited amounts from fruits and vegetables, and supplemented with low‐Phe amino acid (AA) medical foods or glycomacropeptide (GMP) medical foods to provide the majority of dietary nitrogen and micronutrients (Robert et al. 2013; Evans et al. 2014; Singh et al. 2014; Ney et al. 2016; Singh et al. 2016; Pinto et al. 2017; Stroup et al. 2017, 2017). Evidence in humans with PKU and the *Pah^enu2^* murine model of *Pah* deficiency shows an association between long‐term adherence to a low‐Phe diet supplemented with AAs and the presence of chronic kidney disease (Hennermann et al. 2013; Stroup et al. 2017; Burton et al. 2018), as well as skeletal fragility characterized by low bone mineral density (BMD) and increased risk of fractures (Modan‐Moses et al. 2007; Groot et al. 2012; Hansen and Ney, 2014; Coakley et al. 2016; Choukair et al. 2017).

Our research indicates that the standard low‐Phe, high‐acid AA diet used in studies with the *Pah^enu2^* murine model of *Pah* deficiency induces metabolic stress and increases renal workload based on greater renal mass, fluid intake, and polyuria in both *Pah^−/−^* and wild‐type (WT) mice (Solverson et al. 2012). In addition, femurs from *Pah^−/−^* mice are weaker and show lower bone mineral content (BMC) than femurs from WT mice, and this bone phenotype is exacerbated by the low‐Phe, high‐acid AA diet (Solverson et al. 2012). In eight human subjects with PKU, we demonstrated threefold greater renal net acid excretion with ingestion of high‐acid AA medical foods, which could be ameliorated by ingestion of the low‐acid GMP medical foods (Stroup et al. 2017). In this same study, proteinuria and hyperexcretion of urinary creatinine were observed in 38–63% of subjects with PKU with self‐reported lifelong compliance with high‐acid AA medical foods (Stroup et al. 2017). Consistent with this evidence of renal dysfunction in humans with PKU, reduced glomerular filtration rate (GFR) and increased urinary calcium excretion were correlated with graduated increases in ingestion of elemental AAs from medical foods in 67 adolescents and adults with PKU (Hennermann et al. 2013). A retrospective case‐controlled study in the United States found significantly greater prevalence ratios of renal insufficiency (with and without hypertension) and osteoporosis in 3691 subjects with PKU compared to 18,455 controls (Burton et al. 2018). Together, these studies suggest that renal dysfunction is a significant complication for individuals with PKU compliant with AA medical foods, but the relative contributions of the PKU genotype, hyperphenylalaninemia, dietary protein source (intact protein vs. AAs), and dietary acid load remain unclear.

Long‐term ingestion of a high dietary acid load, especially with a concomitant decline in renal function as observed in the elderly population, contributes to osteopenia and osteoporosis (Jehle et al. 2006; Moseley et al. 2012; Jehle et al. 2013). One underlying mechanism for this response is skeletal buffering of an acid load to maintain systemic pH homeostasis (Remer and Manz, 1995; Lemann et al. 2003; Remer et al. 2011). Our objective was to investigate the impact of diets differing in dietary protein source, Phe content, and acid load on bone and renal outcomes in male and female *Pah^−/−^* and WT mice. By reducing the dietary acid load with the buffered AA (BAA) diet, we successfully reduced renal net acid excretion, but did not observe significant improvements in overall bone and renal status.

## Methods

### Animals and experimental design

**Figure 1:**
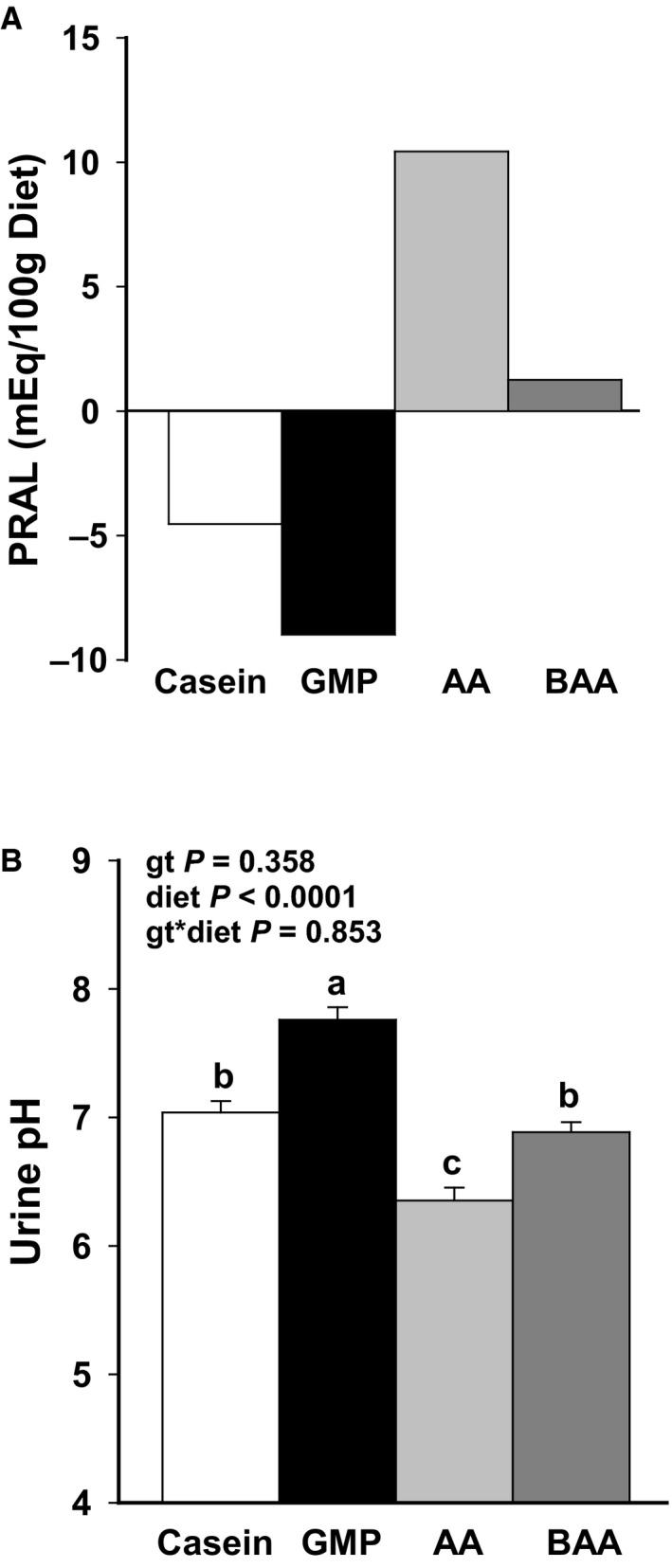
Calculated potential renal acid load (PRAL) (A) of high‐Phe, low‐acid casein diet; low‐Phe, low‐acid glycomacropeptide (GMP) diet; low‐Phe, high‐acid amino acid (AA) diet; and low‐Phe, low‐acid buffered amino acid (BAA) diet. Urine pH (B) of male mice fed the diets for 16–18 weeks. Consistent with the calculated PRAL, ANOVA showed significant effects for only diet to alter pH. Buffering the AA diet was effective in increasing urine pH to the level observed in mice fed the casein control diet. Values are means ± SE for diet main effects; *n* = 12–15. ^a,b,c^Means with different superscript letters indicate significant differences in urine pH due to diet (*P* < 0.01).

The University of Wisconsin‐Madison Institutional Animal Care and Use Committee approved the facilities and protocols used in this study. Experimental animals were produced from a breeding colony (Strain: B6.BTBR‐*Pah^enu2^*/MalnJ*)* by intercrossing *Pah* ^+/−^ mice on the C57BL/6J background to yield *Pah^−/−^* mice (*Pah^enu2/enu2^*) and *Pah* ^+/+^ (WT) mice (Shedlovsky et al. 1993). Experimental mice were genotyped for the *Pah^−/−^* mutation. The experiment utilized a 2 × 2 × 4 factorial design to control for sex (male or female), genotype (PKU, *Pah^−/−^*, or WT, *Pah^+/+^*), and diet (low‐Phe, high‐acid AA, TD.09667; low‐Phe, low‐acid BAA, TD.130407; low‐Phe, low‐acid GMP, TD.120645; high‐Phe, low‐acid casein, TD.09669). The casein diet served as the control diet. The experimental and controls diets were formulated and prepared by Envigo Teklad (Madison, WI). The AA contents of the diets were analyzed by Agriculture Experimental Station Chemical Laboratories at the University of Missouri‐Columbia using cation‐exchange chromatography (cIEC‐HPLC) coupled with post‐column ninhydrin derivatization and quantitation. The mineral contents of the diets were analyzed by Covance Laboratories (Madison, WI) using inductively coupled plasma emission spectrometry for elements (Methods: 984.27, 985.01) and chloride (Methods: 963.05, 971.27, 986.26) as recommended by the Association of Official Agricultural Chemists International. The AA and mineral content of the analyzed diets are described in Tables 1 and 2, respectively. Calculation of the potential renal acid load of the four diets, as previously described (Stroup et al. 2017), suggested that the dietary acid load of the BAA diet was reduced to nearly neutral compared to the high‐acid AA diet (Fig. 1A); the casein diet was neutral and GMP diet was alkaline. Urine pH was measured with a 20‐*µ*L aliquot of urine on a colorimetric pH‐indicator strip (Fluka Analytical, Sigma‐Aldrich, Inc.) in a preliminary study that confirmed the calculated potential renal acid loads of the diets (Fig. 1B).

**Table 1:** Amino acid composition of the diets.

Amino acid, W/W%	Diet			
	Casein	GMP	AA	BAA
Alanine	0.56	0.92	0.26	0.35
Arginine	0.63	0.40	0.97	0.92
Aspartic Acid	1.28	1.58	0.89	0.92
Cysteine	0.31	0.14	0.29	0.26
Glutamic Acid	3.83	3.19	3.60	3.73
Glycine	0.35	0.23	2.34	2.23
Histidine	0.55	0.37	0.31	0.35
Isoleucine	0.93	1.50	0.74	0.70
Leucine	1.71	1.09	1.05	1.09
Lysine	1.45	1.19	1.39	1.46
Methionine	0.48	0.89	0.61	0.29
Phenylalanine	0.92	0.22	0.23	0.21
Proline	1.87	1.70	0.33	0.34
Serine	0.95	1.04	0.26	0.28
Taurine	0.10	0.10	0.02	0.01
Threonine	0.77	2.25	0.77	0.74
Tryptophan	0.24	0.16	0.19	0.19
Tyrosine	0.77	0.45	0.39	0.40
Valine	1.17	1.27	0.79	0.80

Amino acids contents of the diets were analyzed by the Agriculture Experimental Station Chemical Laboratories at the University of Missouri‐Columbia. AA, amino acid; BAA, buffered amino acid; GMP, glycomacropeptide.

**Table 2:** Mineral composition of the diets.

Minerals, mg/100 g diet	Diet			
	Casein	GMP	AA	BAA
Calcium	531	518	551	571
Copper	0.86	2.78	0.73	0.50
Iron	4.63	9.58	4.47	4.70
Magnesium	46.3	80.7	44.6	74.2
Manganese	1.01	0.96	1.03	0.90
Phosphorus	319	308	317	340
Potassium	406	457	424	438
Sodium	480	476	472	341
Zinc	3.66	3.87	3.10	8.80
Chloride	418	262	950	508

Mineral analyses of the diets were conducted by Covance Laboratories (Madison, WI). AA, amino acid; BAA, buffered amino acid; GMP, glycomacropeptide.

Male (*n* = 108) and female (*n* = 104) mice were weaned at 21 days of age, separated by sex and housed in shoebox cages, and randomized to a diet. Mice had unrestricted access to food and water. The facility maintained a 12:12‐h light‐dark cycle, temperatures between 20 and 25.5°C, and 30–40% humidity. Mice were fed the experimental diets from weaning through young adulthood (23–27 weeks of age), which resulted in mice being fed diet for an average of 21.5 ± 0.06 weeks before euthanasia (*n* = 212 mice; 24–29 mice per diet).

After consuming the experimental diet for 16–18 weeks, mice were placed in metabolic cages for 72 h for continuous surveillance of food and water intake and urine output. At the same time each morning, the following parameters were assessed: body weight, food intake, water intake, urine volume, and urine pH. Urine samples were stored at −20°C and concentrations of renal net acid and ammonium in urine were measured using titrations as previously described (Stroup et al. 2017).

At the end of the study, body weights were obtained prior to isoflurane anesthesia and euthanasia by exsanguination. Blood, brain, liver, kidneys, spleen, small intestine, cecum, colon, and femurs were collected. Tails were obtained from female WT and *Pah^−/−^* mice fed the AA, BAA, GMP, or casein diets (*n* = 4/genotype). Syringes used to collect blood were first rinsed with 5% EDTA solution prior to collection to prevent coagulation. Organ and cecal content weights were obtained. Organs were wrapped in aluminum foil, snap‐frozen in liquid nitrogen, and stored at −80°C; kidneys were placed into 10% neutral buffered formalin or snap‐frozen. Cecal contents were stored at −80°C. Femurs and tails were wrapped in phosphate buffered saline‐saturated gauze and stored at −80°C and −20°C, respectively.

### Renal histology and immunohistochemistry

One kidney from each mouse was bisected longitudinally and fixed in 10% buffered formalin. Kidneys fixed in formalin were processed and embedded in paraffin using standard techniques, sectioned at 5 *µ*m, and stained with hematoxylin and eosin (H&E) or periodic acid–Schiff (PAS). Frozen kidneys were cryosectioned at 7 *µ*m and stained with Oil Red O.

Cortical tubular vacuolation, medullary tubular dilation, and proteinosis were identified and graded for severity by a boarded pathologist with expertise in murine renal pathology. Severity scores were based on a six‐level scale of absent (Grade 0), minimal (Grade 1), mild (Grade 2), moderate (Grade 3), marked (Grade 4), and severe (Grade 5) (Holland, 2005). Medullary tubular dilation severity scoring adhered to the following: Grade 0 = absent (no dilation); Grade 1 = minimal (one dilated medullary tubule); Grade 2 = mild (two to three dilated medullary tubules); Grade 3 = moderate (four to five dilated medullary tubules); Grade 4 = marked (six to seven dilated medullary tubules); and Grade 5 = severe (eight or more dilated medullary tubules).

To further identify the vacuolated renal cortical tubular epithelial cells, immunohistochemistry of renal sections was performed with the anti‐aquaporin 1 antibody (ab15080, Abcam, Cambridge, MA) using published protocols (Bauchet et al. 2011).

### Assessment of femoral biomechanics and bone mineral content

Prior to measurement of mineral content and biomechanical analysis, femurs were gradually warmed by placing them at 4°C for 12 h, and then allowing them to come to room temperature prior to analysis. Ex vivo dual‐energy x‐ray absorptiometry (DXA) with PIXImus software version 2.10 (GE/Lunar Corp, Madison, WI) was performed on femurs to obtain BMD and BMC. We tested femoral diaphysis biomechanical performance by quasi‐static 3‐point bending under displacement control with a support span of 7.5 mm as previously described (Solverson et al. 2012).

### Evaluation of posttranslational modifications of collagen

Collagen was extracted and digested as previously described from whole tails and femurs (Pokidysheva et al. 2013). To evaluate glycosylated hydroxylysine residues at the A1 site (Pro‐986) and A3 site (Pro‐707) of the α1‐chain of type 1 collagen, hydrazide chemistry was used to isolate the glycosylated peptides, and MS/MS spectra were examined for peaks of interest (Pokidysheva et al. 2013). To quantify collagen crosslinking and glycosylation in collagen obtained from the femur, tendon, and tail, samples were subjected to acid and alkaline hydrolysis and analyzed using the Hitachi L‐8800 amino acid analyzer (San Jose, CA).

### Statistical analysis

Male and female mice were analyzed separately due to the large interaction of sex with outcome variables. Data were analyzed in SAS 9.4 using two‐way ANCOVA with a covariate for body weight or two‐way ANOVA (if the covariate for body weight was not significant) within PROC GLM (SAS Institute Inc., 2007, Cary, NC) to test for main effects due to genotype (*Pah^−/−^* or WT), and diet (AA, BAA, casein, GMP), and genotype by diet interaction. Fisher's least significant difference test was used to detect differences among groups. If statistical assumptions were not met after the log transformation was performed, then rank variables from dependent variables were calculated using PROC RANK. Then, using PROC GLM, ANOVA was performed with the rank values. Changes in body weight were analyzed using a repeated measures model within PROC MIXED. Log transformations were performed to meet assumptions of equal variance and normality as needed. Data in figures are presented as means ± SE. Figures show the data for all eight groups (two genotypes: WT and *Pah^−/−^*; four diets: casein, AA, BAA, and GMP) when the genotype by diet interaction was significant. If the genotype by diet interaction was not significant, then the data in the figures show the main effects only. *P*‐values < 0.05 were considered significant.

## Results

### Body weight, food consumption, and organ weights

**Figure 2:**
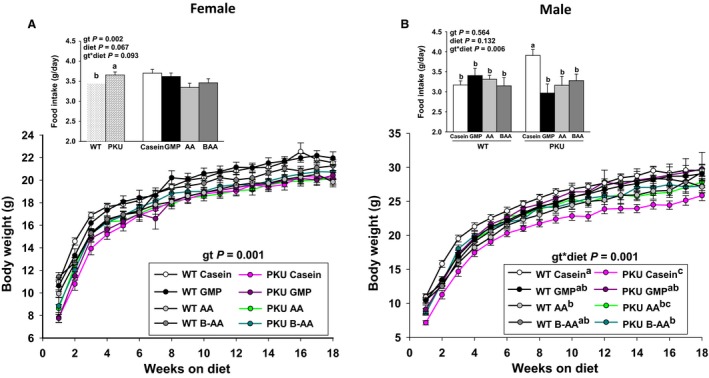
Change in body weight and food intake of female (A) and male (B) phenylketonuria (PKU, *Pah^−/−^*) and wild‐type (WT) mice fed casein, glycomacropeptide (GMP), amino acid (AA), or buffered AA (BAA) for 18 weeks, from 3 week through 21 week of age. Repeated measures analysis was used to analyze changes in body weight over time and ANCOVA with a covariate for body weight was used to analyze food intake. In female mice, there was a significant effect for genotype (gt) such that PKU mice ate more food, but showed reduced growth compared to WT mice (A). In male mice, there was a significant gt x diet interaction for food intake and growth such that PKU mice fed the high‐Phe casein diet demonstrated greater food intake, but showed reduced growth compared to all four WT groups (B). Values are means ± SE; *n* = 8–12 mice per treatment group. ^a,b,c^Means with different superscript letters indicate significant differences (*P* < 0.05).

Growth and food intake of mice from weaning at 3 weeks through 21 weeks of age are shown in Figure 2. Female *Pah^−/−^* mice consumed significantly more food than female WT mice based on the data collected in metabolic cages between 16 and 18 weeks of age for one 24‐hour time period. However, female *Pah^−/−^* mice showed reduced growth from 3 to 21 weeks of age compared with WT mice. Male *Pah^−/−^* mice showed increased food intake with the casein diet, but reduced growth compared to all four male WT groups. Overall, regardless of diet, final body weights were 5–10% lower in *Pah^−/−^* versus WT mice.

The casein control diet provided a high‐Phe content (0.92 g Phe/100 g diet) compared with the AA and GMP diets (0.21–0.23 g Phe/100 g diet, Table 1). Plasma Phe concentrations in *Pah^−/−^* and WT mice fed the same casein, AA, and GMP diets were reported in two previous studies and were not determined in the present study (Solverson et al. 2012; Solverson et al. 2012). In summary, ingestion of the casein diet resulted in a plasma Phe concentration of 2103 ± 92 *µ*mol/L in *Pah^−/−^* mice and 51 ± 2 *µ*mol/L in WT mice. In *Pah^−/−^* mice, ingestion of the low‐Phe AA and GMP diets resulted in plasma Phe concentrations of 766 ± 26 *µ*mol/L and 766 ± 18 *µ*mol/L, respectively (Solverson et al. 2012). Plasma Phe concentrations for mice fed the BAA diet in the current study were likely similar to that observed with ingestion of the AA and GMP diets because food intake was not significantly different and the Phe contents of all three diets were similar (0.21–0.23 g Phe/100 g diet). Moreover, growth was not significantly different in male and female WT and *Pah^−/−^* mice fed the AA and BAA diets (Fig. 2).

The effects of the dietary treatments on relative organ weights (g/100 g body weight) are shown in Table 3. Regardless of sex, relative liver weights were significantly greater in *Pah^−/−^* versus WT mice. Additionally, relative liver weights were significantly greater with ingestion of the casein compared with the AA diet in *Pah^−/−^* mice.

**Table 3:** Organ weights of female and male wild‐type (WT) and *Pah^−/−^* mice fed casein, amino acid, buffered amino acid, and glycomacropeptide diets.

Organ	WT				*Pah^−/−^*				*P* Value		
	Casein	GMP	AA	BAA	Casein	GMP	AA	BAA	gt	diet	gt*diet
Females											
Liver, g/100 g bw	4.45 ± 0.08^b^	4.54 ± 0.04^b^	4.44 ± 0.08^b^	4.61 ± 0.05^b^	5.00 ± 0.15^a^	4.73 ± 0.07^ab^	4.47 ± 0.22^b^	5.00 ± 0.10^a^	0.0005	0.02	0.12
Spleen, g/100 g bw	0.46 ± 0.02^cd^	0.44 ± 0.02^d^	0.47 ± 0.03^cd^	0.44 ± 0.02^d^	0.58 ± 0.04^b^	0.46 ± 0.02^cd^	0.65 ± 0.03^a^	0.51 ± 0.03^c^	<0.0001	<0.0001	0.01
Brain, g/100 g bw	1.82 ± 0.03^bc^	1.81 ± 0.03^bcd^	1.96 ± 0.05^a^	1.86 ± 0.03^ab^	1.63 ± 0.07^e^	1.72 ± 0.04^cde^	1.70 ± 0.05^de^	1.64 ± 0.05^e^	<0.0001	0.09	0.24
Small Intestines, mg/cm	3.06 ± 0.05^abc^	2.98 ± 0.04^cd^	3.17 ± 0.04^a^	3.15 ± 0.05^ab^	2.92 ± 0.06^d^	2.98 ± 0.05^cd^	3.03 ± 0.02^bcd^	3.16 ± 0.05^ab^	0.048	0.0003	0.19
Colon, mg/cm	19.4 ± 0.72^b^	21.2 ± 0.77^ab^	21.3 ± 0.82^ab^	21.0 ± 0.61^ab^	20.9 ± 0.66^ab^	20.0 ± 0.52^ab^	21.3 ± 0.69^a^	22.1 ± 1.21^ab^	0.48	0.50	0.37
Cecum Content, g	0.15 ± 0.01^c^	0.23 ± 0.03^ab^	0.17 ± 0.01^c^	0.16 ± 0.01^c^	0.20 ± 0.03^bc^	0.30 ± 0.05^a^	0.20 ± 0.02^bc^	0.18 ± 0.02^bc^	0.03	0.0001	0.89
Males											
Liver, g/100 g bw	4.33 ± 0.11^cd^	4.59 ± 0.10^bc^	4.23 ± 0.10^d^	4.37 ± 0.12^cd^	5.00 ± 0.16^a^	4.62 ± 0.13^bc^	4.42 ± 0.12^cd^	4.85 ± 0.17^ab^	0.0002	0.04	0.049
Spleen, g/100 g bw	0.37 ± 0.02^bc^	0.31 ± 0.02^d^	0.32 ± 0.01^cd^	0.34 ± 0.02^bcd^	0.46 ± 0.03^a^	0.35 ± 0.02^bcd^	0.38 ± 0.02^b^	0.37 ± 0.01^bc^	0.0001	0.0006	0.56
Brain, g/100 g bw	1.27 ± 0.03^c^	1.39 ± 0.04^ab^	1.49 ± 0.03^a^	1.34 ± 0.05^bc^	1.29 ± 0.03^bc^	1.32 ± 0.04^bc^	1.31 ± 0.03^bc^	1.32 ± 0.05^bc^	0.03	0.02	0.07
Small Intestines, mg/cm	2.92 ± 0.05^cde^	2.92 ± 0.04^cde^	3.01 ± 0.03^abc^	3.14 ± 0.05^a^	2.88 ± 0.06^de^	2.85 ± 0.06^e^	2.99 ± 0.04^bcd^	3.13 ± 0.06^ab^	0.25	<0.0001	0.95
Colon, mg/cm	21.1 ± 0.66^ab^	22.7 ± 0.68^a^	19.7 ± 0.59^b^	20.4 ± 1.27^ab^	21.5 ± 0.75^ab^	22.2 ± 0.76^ab^	21.3 ± 0.80^ab^	22.2 ± 1.10^ab^	0.19	0.20	0.57
Cecum Content, g	0.23 ± 0.03^bcd^	0.35 ± 0.02^a^	0.25 ± 0.02^bc^	0.18 ± 0.02^d^	0.22 ± 0.01^bcd^	0.35 ± 0.03^a^	0.27 ± 0.03^b^	0.20 ± 0.02^cd^	0.39	<0.0001	0.86

Values are means  ± SE. Sample sizes are 10–16 mice per group. Statistical analysis included ANOVA with effects for genotype (gt), diet, and gt x diet interaction. Separate analyses were performed for female and male mice. AA, amino acid; BAA, buffered amino acid; bw, body weight; genotype, gt; and GMP, glycomacropeptide.

Relative spleen weights were also significantly greater in *Pah^−/−^* compared with WT mice. In male WT and *Pah^−/−^* mice, relative spleen weights were significantly greater with ingestion of the casein diet compared to the AA, BAA, and GMP diets (statistical main effect of diet). In female *Pah^−/−^* mice, relative spleen weights were significantly different among all groups, with mice fed the AA diet having greater relative spleen weights than mice fed the casein, BAA, or GMP diets. Mice fed the BAA and GMP diets demonstrated similar relative spleen weights to WT control mice. Regardless of sex, relative brain weights were greater in WT compared with *Pah^−/−^* mice.

Relative small intestine weights (mg/cm small intestine) were significantly greater with both the AA and BAA diets compared with the casein and GMP diets. Cecal content weights were significantly greater in mice that ingested the GMP diet compared to the casein, AA, and BAA diets; this may be partially attributed to the prebiotic effects of GMP (Sawin et al. 2015; Ntemiri et al. 2017). Relative colon weights (mg/cm colon) did not differ across groups.

### Renal status

**Figure 3:**
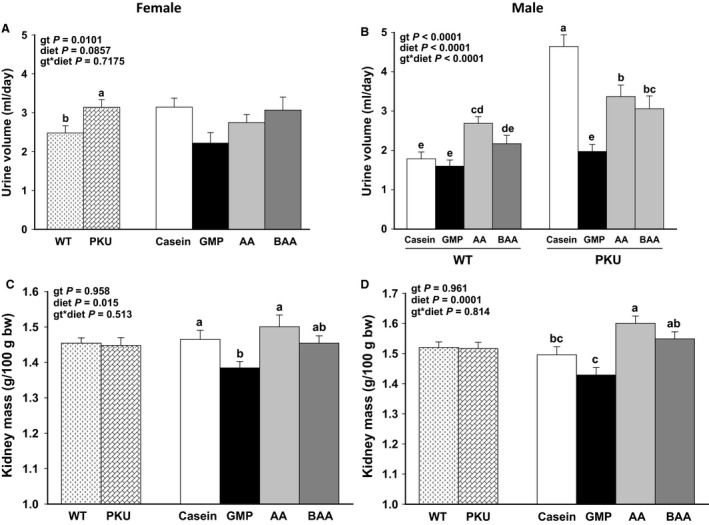
Urine volume (A female, B male) and kidney mass (C female, D male) of phenylketonuria (PKU, *Pah^−/−^*) and wild‐type (WT) mice fed casein, glycomacropeptide (GMP), amino acid (AA), or buffered AA (BAA). For female mice, figures show ANOVA significant main effects for only genotype (gt) to impact urine volume (A) and for only diet to impact kidney mass (C). Female PKU mice had increased urine volume compared with female WT mice and increased kidney mass with the casein and AA diets compared with the GMP diet. For male mice, figures show ANOVA significant main effects for genotype by diet interaction with data presented for all eight treatment groups for urine volume (B) and for only the diet main effect to impact kidney mass (D). Male PKU mice fed the casein, AA, or BAA diets had significantly higher urine volume compared to male PKU mice fed the GMP diet and WT mice fed the casein, GMP, and BAA diets. Kidney mass was significantly greater with the AA and BAA diets compared with the GMP diet for male mice. Regardless of sex or gt, the BAA diet not significantly reduce urine volume or kidney mass compared to the AA diet. Values are means ± SE; *n* = 8–14 mice per treatment group. ^a,b,c,d,e^Means with different superscript letters indicate significant differences due to gt, diet, or the gt x diet interaction (*P* < 0.05).

Regardless of genotype, relative kidney weights were significantly greater in male and female *Pah^−/−^* mice that ingested the high‐acid AA diet than the low‐acid GMP diet (Fig. 3). The low‐acid BAA diet did not alter relative kidney weights or urine volume as compared with the high‐acid AA diet. Urine volume was significantly greater in male WT mice fed the high‐acid AA diet than in male WT mice fed the low‐acid casein and low‐acid GMP diets, whereas in male *Pah^−/−^* mice the high‐Phe, low‐acid casein diet showed significantly higher urine volume compared to high‐acid AA, low‐acid BAA, and low‐acid GMP diets (Fig. 3). Absolute kidney weights ranged from 0.30 to 0.31 g for female mice and 0.41 to 0.44 g for male mice, but did not demonstrate a significant diet effect. This confirmed the importance of relative weights due to the significantly different growth curves in *Pah^−/−^* versus WT mice. Regardless of genotype, the low‐acid GMP diet demonstrated the lowest urine volume in male WT and *Pah^−/−^* mice and was not significantly different from the urine volume for the male WT mice fed the low‐acid casein diet.

**Figure 4:**
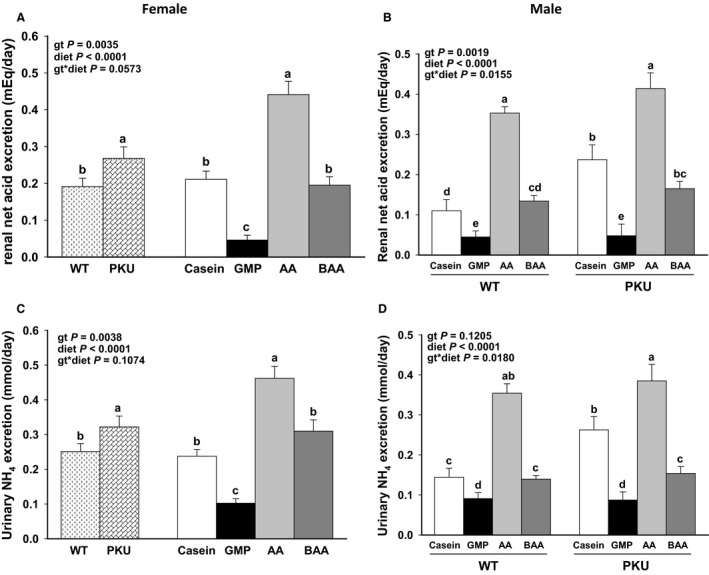
Renal net acid excretion (A female, B male) and urinary ammonia excretion (C female, D male) of phenylketonuria (PKU, *Pah^−/−^*) and wild‐type (WT) mice fed casein, glycomacropeptide (GMP), amino acid (AA), or buffered AA (BAA). For female mice, figures show ANOVA significant main effects for both genotype (gt) and diet without interaction to impact renal net acid excretion (A) and urinary ammonium excretion (C). Female PKU mice had increased excretion of renal net acid and ammonium compared with female WT mice. For male mice, figures show ANOVA significant main effects for gt x diet interaction with data presented for all eight treatment groups for renal net acid excretion (B), and urinary ammonium excretion (D). Regardless of sex or gt, the BAA diet significantly reduced the excretion of renal net acid and ammonium compared to the AA diet with the lowest excretion observed in mice fed the GMP diet. Values are means ± SE; *n* = 8–14 mice per treatment group. ^a,b,c,d^Means with different superscript letters indicate significant differences due to gt, diet, or gt × diet interaction (*P *< 0.05).

Regardless of sex or genotype, the low‐acid BAA diet significantly reduced the excretion of renal net acid and ammonium by ~50% as compared to the high‐acid AA diet (Fig. 4). Renal excretion of net acid did not differ in *Pah^−/−^* mice fed the BAA and casein diets. Consistent with lower relative kidney weights and urine volume, excretion of net acid and ammonium was significantly lower in mice from all treatment groups fed the low‐acid GMP diet compared to the other three diets.

### Renal histology

**Figure 5:**
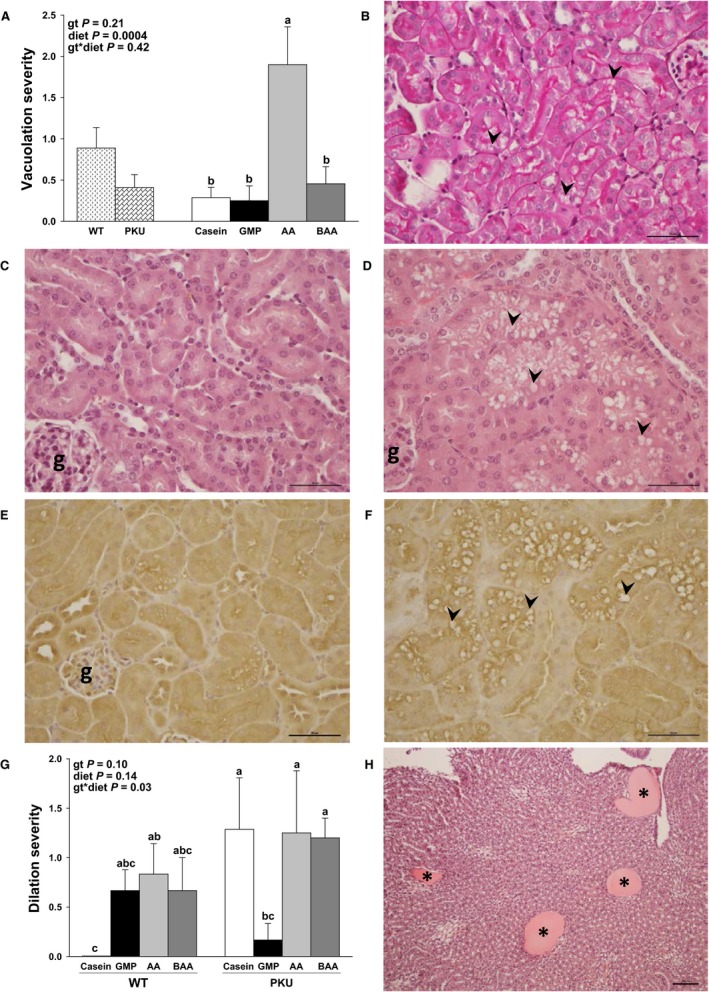
Renal histology in male WT and *Pah^−/−^* (PKU) mice fed casein, GMP, AA, and BAA diets. Statistical analysis included two‐way ANOVA with main effects for genotype (gt, WT or PKU), diet (casein, AA, BAA, or GMP), and (gt) x diet interaction. Superscript letters that differ among groups are significantly different (*P *< 0.05). Values are means ± SE; *n* = 4–7 per genotype per diet. ^a,b,c^Means with different superscript letters indicate significant differences due to gt, diet, or gt x diet interaction (*P* < 0.05). (A) Regardless of gt, mice fed the high‐acid AA diet demonstrated greater multifocal cortical tubular vacuolation severity (diet, *P *= 0.0004). (B) Periodic acid‐Schiff (PAS) staining of the renal cortex fails to identify polysaccharides within the vacuolated cortical tubular epithelial cells (arrowheads), yet positively stains the tubular basement membranes (internal positive control) in a male WT mouse fed the AA diet. (C) Normal renal cortical tubular epithelium in a male WT mouse fed the amino acid diet. D. Vacuolation (arrowheads) of renal cortical tubular epithelial cells in a male WT mouse fed the AA diet. (E) Aquaporin‐1 immunohistochemistry identifies the stronger‐staining epithelium as the proximal convoluted tubule in the kidney of a male PKU mouse fed the AA diet. F. Aquaporin‐1 immunohistochemistry stains the vacuolated epithelial cells in a male WT mouse fed the AA diet, identifying the site of vacuolation as proximal convoluted tubules. (G) WT and PKU mice fed the AA diet and PKU mice fed casein and BAA diets show greater medullary tubular dilation severity compared to WT mice fed the casein diet (control) and PKU mice fed the GMP diet (gt x diet interaction, *P *= 0.03)*.* (H) Photomicrographs of H&E stained kidney showing moderate (Grade 3) dilation and possible proteinosis (asterisks) in a male PKU mouse fed the AA diet. 5B, 5H scale bar = 100 *µ*m. 5C–F, scale bar = 50 *µ*m. AA, amino acid; BAA, buffered amino acid; g, glomerulus; GMP, glycomacropeptide; H&E staining; hematoxylin and eosin staining; PKU, phenylketonuria; WT, wild‐type.

**Figure 6:**
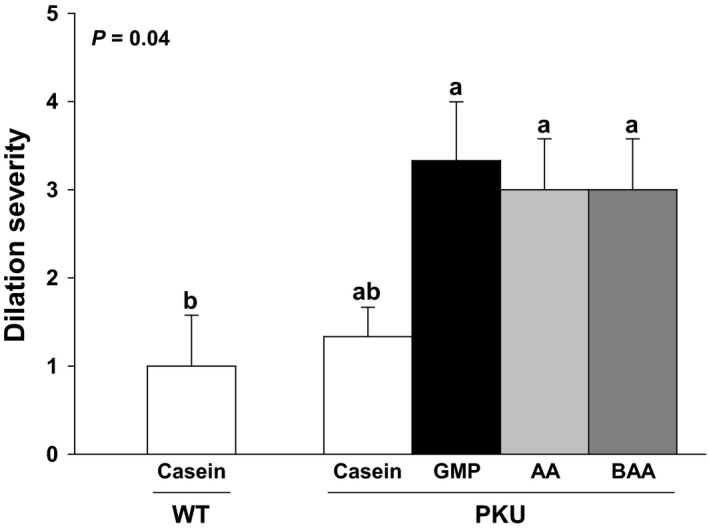
Greater medullary tubular dilation severity was observed in female *Pah^−/−^* (PKU) mice fed the glycomacropeptide (GMP), amino acid (AA), and buffered AA (BAA) diets compared to female wild‐type (WT) mice fed the casein diet (control). Statistical analysis included one‐way ANOVA. Values are means ± SE; *n* = 3/group. ^a,b^Means with different superscript letters indicate groups that are significantly different (*P* < 0.05).

Given the clear effect of diet on renal mass and urine volume, histology was performed on renal sections to assess microarchitecture in male and female WT and *Pah^−/−^* mice (Fig. 5, 6). Regardless of genotype, male mice fed the high‐acid AA diet exhibited multiple areas of mild cortical tubular vacuolation, whereas male mice fed the low‐acid casein, BAA, and GMP diets had absent or minimal tubular vacuolation (Fig. 5A, C and D). Cortical tubular vacuolation was not identified in female mice regardless of genotype or diet (data not shown). Vacuoles consisted of variably sized, clear intracytoplasmic structures (Fig. 5B and D). These vacuoles failed to show PAS staining, suggesting that they do not contain polysaccharides (Fig. 5B). Oil Red O staining of frozen sections also failed to demonstrate the presence of lipid or compressed cytoplasm within vacuoles (data not shown). To confirm that the tubular vacuolation observed in male mice was located within the proximal convoluted tubules, immunohistochemistry with aquaporin‐1 was performed. The aquaporin‐1 protein is specifically expressed within proximal convoluted tubular epithelial cells. Identification of vacuoles within tubules with positive aquaporin‐1 staining indicates that vacuoles accumulate within the proximal convoluted tubules (Fig. 5E and F). Vacuolation of proximal convoluted tubular epithelial cells suggests proximal tubular dysfunction in male mice fed high‐acid AA diet.

Medullary tubular dilation consisted of widened tubules in the medulla filled with brightly eosinophilic (proteinaceous) fluid (Fig. 5H). In general, minimal to mild (Grades 1–2) medullary tubular dilation was observed in male WT and *Pah^−/−^* mice fed the high‐acid AA and low‐acid BAA diets, and in male *Pah^−/−^* mice fed the low‐acid, high‐Phe casein diet (Fig. 5G). Although medullary dilation ranged from absent (Grade 0) to moderate (Grade 3) in male mice, moderate medullary tubular dilation was observed in three of 22 male *Pah^−/−^* mice, whereas moderate tubular dilation was not observed in male WT mice. In contrast, six of 12 female *Pah^−/−^* mice fed AA, BAA, or GMP diets demonstrated moderate (Grade 3) medullary tubular dilation compared to WT female mice fed the casein diet (Fig. 6). The brightly eosinophilic material seen in the dilated medullary tubules of male and female *Pah^−/−^* mice is likely proteinaceous fluid, based on the homogenous size of the vacuoles, lack of compressed local cytoplasm, and negative PAS and Oil Red O staining (Fig. 5H). The presence of protein within the medullary tubules may be due to the failure of the proximal convoluted tubules to re‐uptake protein filtered by the glomeruli. There was no light microscopic evidence of glomerular dysfunction in this study.

Polyuria refers to excessive urinary excretion and is a functional manifestation of renal dysfunction. According to the Jax Phenome Database, the average urinary excretion of C57BL/6J mice is 1.6 mL/day (Smith et al. 2018). Increased urine excretion was noted in male WT and *Pah^−/−^* mice fed the AA and BAA diets and in male *Pah^−/−^* mice fed the casein diet. Male *Pah^−/−^* mice fed the casein diet produced more urine (4.64 ± 0.30 mL/day) than male *Pah^−/−^* mice fed the AA diet (3.37 ± 0.32 mL/day), than male *Pah^−/−^* mice fed the BAA diet (3.06 ± 0.32 mL/day), than male *Pah^−/−^* mice fed the GMP diet (1.97 ± 0.18 mL/day) (Fig. 3). Compared to the urine output from male WT mice fed the casein diet (1.78 ± 0.18 mL/day), male WT mice fed the AA diet (2.69 ± 0.17 mL/day) and male WT mice fed the BAA diet (2.17 ± 0.22 mL/day) excreted greater urinary volumes, but not male WT mice fed the GMP diet (1.60 ± 0.16 mL/day).

The dilation of medullary tubules observed in female *Pah^−/−^* mice fed the low‐Phe diets (AA, BAA, GMP, Fig. 6) also indicates tubular dysfunction and likely contributed to the observed excessive urine excretion in the female *Pah^−/−^* mice. Female *Pah^−/−^* mice produced 3.14 ± 0.20 mL urine/day, while female WT mice produced 2.48 ± 0.19 mL urine/day (Fig. 3). Interestingly, female *Pah^−/−^* and WT mice fed the casein diets demonstrated mild tubular dilation.

### Femoral mineralization and strength

**Figure 7:**
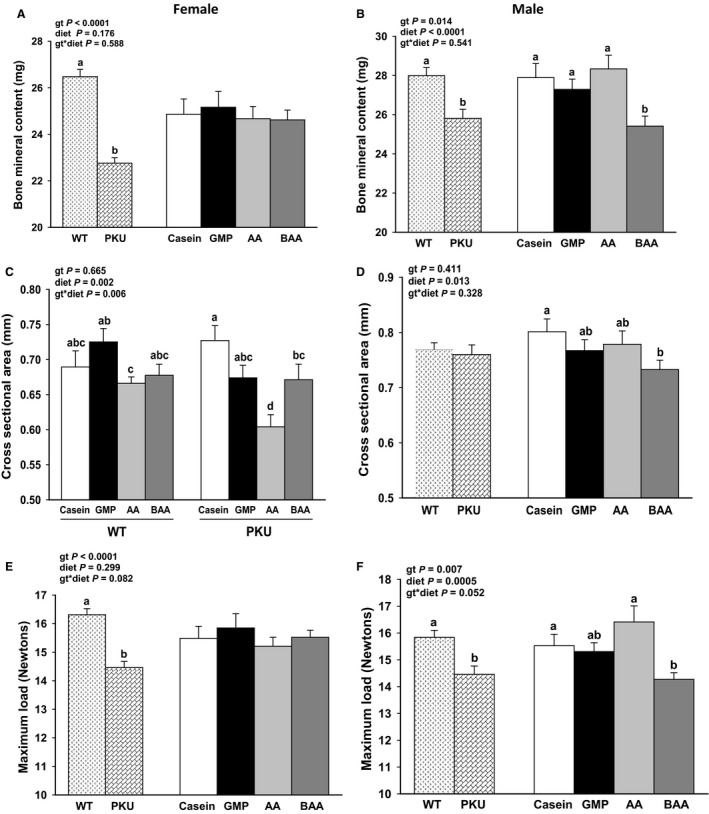
Femoral measures in female (A, C, and E) and male (B, D, and F) phenylketonuria (PKU, *Pah^−/−^*) and wild‐type (WT) mice fed casein, glycomacropeptide (GMP), amino acid (AA), or buffered AA (BAA) diets. All data analyzed separately for males and females by ANCOVA with a covariate for body weight. Data are presented for all eight groups when significant genotype (gt) × diet interaction was observed and data are presented as main effects for gt and diet when there was no significant interaction. Regardless of sex and diet, PKU mice displayed significantly lower femoral bone mineral content and tolerated a lower maximum load before breaking compared to WT mice; this effect of the PKU gt to impair bone status was more pronounced in female compared with male mice. Values are means ± SE; *n* = 10–15 for femoral measures per treatment group. ^a,b,c,d^Means with different superscript letters indicate significant differences due to gt, diet, or the gt x diet interaction (*P* < 0.05).

The *Pah^−/−^* genotype and sex showed differential effects on femoral mineralization, cross‐sectional area, and maximum load tolerated before fracture (Fig. 7). Male *Pah^−/−^* mice showed significantly lower femoral BMC and maximum load tolerated before femoral breaking with the BAA compared with the AA diet (main effect for diet). These data suggest that buffering the AA diet did not improve femoral mineralization or strength in male *Pah^−/−^* mice, in spite of lower excretion of renal net acid and ammonium with the AA diet.

In female mice, femoral BMC and maximum load tolerated before breaking were similar across diets. Femoral cross‐sectional area showed significant genotype by diet interaction such that *Pah^−/−^* mice fed the AA diet had significantly lower cross‐sectional area as compared to *Pah^−/−^* mice fed the BAA, GMP, and casein diets. Female WT mice fed the AA diet had significantly lower femoral cross‐sectional area than female WT mice fed GMP diet.

Regardless of sex and diet, *Pah^−/−^* mice displayed significantly lower femoral BMC and BMD (data not shown) and tolerated a lower maximum load before breaking compared to WT mice (Fig. 7C–D, G–H). These data suggest that *Pah* deficiency impairs bone accretion. The magnitude of decrease in femoral BMC and maximum load was greater in female *Pah^−/−^* compared with male *Pah^−/−^* mice (BMC, percent change: female; *Pah^−/−^* = −14% vs. male; *Pah^−/−^* = −7.75%; Maximum load, percent change: female; *Pah^−/−^* = −11.34% vs. male; *Pah^−/−^* = −8.68%).

### Posttranslational modifications of collagen

Similar collagen crosslinking and glycosylation in collagen obtained from the femur, tendon, and tail in female WT and *Pah^−/−^* mice were observed for all four diets *n* (Table 4). Although collagen was assessed only in female mice with a small sample size (*n* = 4 per genotype), these data suggest that suboptimal skeletal outcomes in *Pah^−/−^* mice are not related to alterations in collagen metabolism. The impact of diet on collagen metabolism in WT and *Pah^−/−^* mice was not assessed due to the small sample size (*n* = 1 per genotype per diet).

**Table 4:** Similar patterns of posttranslational modification of collagen between female wild‐type (WT) and *Pah^−/−^* mice.

	WT	Pah^−/−^	*P* Value
Femur, (3‐Hyp)/(3‐Hyp + Pro)	0.035 ± 0.009	0.050 ± 0.018	0.66
Femur, (4‐Hyp)/(4‐Hyp + Pro)	0.433 ± 0.008	0.425 ± 0.009	0.54
Femur, (OH‐Lys/(OH‐Lys + Lys)	0.223 ± 0.018	0.250 ± 0.012	0.27
Tendon, (3‐Hyp)/(3‐Hyp + Pro)	0.030 ± 0.000	0.025 ± 0.003	0.13
Tendon, (4‐Hyp)/(4‐Hyp + Pro)	0.440 ± 0.010	0.448 ± 0.003	0.87
Tendon, (OH‐Lys/(OH‐Lys + Lys)	0.245 ± 0.035	0.273 ± 0.053	1.00
Femur, GGH (GGH/Val)	0.025 ± 0.004	0.025 ± 0.005	0.80
Femur, GH (GH/Val)	0.046 ± 0.004	0.043 ± 0.006	0.60
Tail, GGH (GGH/Phe)	0.180 ± 0.005	0.170 ± 0.006	0.51
Tail, GH (GH/Phe)	0.244 ± 0.005	0.239 ± 0.006	0.90

To evaluate collagen crosslinking and glycosylation in collagen obtained from the femur, tendon and tail, samples were subjected to acid and alkaline hydrolysis and analyzed using an amino acid analyzer. Values are means ± SE, *n* = 4/genotype. One‐way ANOVA with a main effect for genotype was performed. GH, galactosylhydroxylysine; GGH, glucosylgalactosylhydroxylysine; Hyp, hydroxyproline; Lys, lysine; OH‐Lys, hydroxylysine; Phe, phenylalalnine; Pro, proline; Val, valine; WT, wild type.

## Discussion

Renal dysfunction and osteoporosis are complications of PKU for which the pathogeneses are poorly understood (Hennermann et al. 2013; Hansen and Ney, 2014; Burton et al. 2018). We hypothesized that renal dysfunction and osteoporosis in PKU may be partially due to long‐term ingestion of a high dietary acid load derived from elemental AAs when consumed as the primary dietary protein source. This hypothesis is supported by the evidence that long‐term ingestion of a high dietary acid load requires renal and skeletal buffering to maintain acid–base homeostasis, thereby increasing urinary excretion of renal net acid and promoting bone resorption (Lemann et al. 2003; Hennermann et al. 2013; Bounoure et al. 2014). In this study, we demonstrated that the high‐acid AA diet could be buffered by decreasing the content of sulfur‐containing AAs (methionine and cysteine) and chloride as evidenced by the significant reduction in the urinary excretion of renal net acid and ammonium with the low‐acid BAA diet. However, buffering the AA diet did not improve renal and bone outcomes in *Pah^−/−^* mice.

Chronic kidney disease can be a consequence of persistent or progressive damage to renal glomeruli, tubules, interstitium, or vasculature and is characterized by reduced GFR (<90 mL/min), altered renal histological structures, polyuria, and proteinuria (Yang et al. 2010; Hennermann et al. 2013; Tani et al. 2017). The adjusted prevalence ratios for renal insufficiency with and without hypertension in subjects with PKU are estimated to be 2.20 and 1.57, respectively, indicating that chronic kidney disease occurs more frequently in individuals with PKU than the general population (Burton et al. 2018). However, it is unclear whether the increased prevalence of renal insufficiency in PKU is due to an underlying disease mechanism or due to chronic ingestion of elemental AA medical foods containing a high dietary acid load (Stroup et al. 2017).

Dietary intake of AAs induces a greater metabolic workload due to more rapid intestinal absorption and increased hepatic degradation of AAs versus intact protein, thereby increasing renal workload through diuresis for nitrogen detoxification (Heller et al. 1987; Lemann et al. 2003; Bounoure et al. 2014; Tani et al. 2017). A high dietary acid load also increases renal workload as the kidney plays a pivotal role in acid–base homeostasis through renal excretion of renal acid and NH_4_^+^ for removal of metabolic protons and increased synthesis of bicarbonate, in conjunction with urinary excretion of sulfate and urea (Wesson and Simoni, 2010; Wesson et al. 2011; Bounoure et al. 2014; Tani et al. 2017). The only clinical study to have investigated the impact of ingestion of AAs on GFR, which is the gold standard for the assessment of renal function in humans, demonstrated a dose‐dependent reduction in GFR with graduated increases in dietary intake of elemental AAs from medical foods in 67 subjects with PKU (Hennermann et al. 2013). It has been previously reported that the majority of commercially available AA medical foods have a high dietary acid load (Stroup et al. 2017); thus, it is reasonable to assume that the AA medical foods that were used in the clinical study by Hennermann et al. provided a high dietary acid load (Hennermann et al. 2013). In the general population, a high dietary acid load and ingestion of elemental AAs increase renal workload and contribute to chronic kidney disease incidence (Heller et al. 1987; Rebholz et al. 2015; Ney and Etzel, 2017).

Despite the fact that dietary acid load was successfully reduced with the low‐acid BAA diet in this murine study, reducing the dietary acid load of the BAA diet did not improve overall renal status and produced different renal outcomes by sex. Sex‐specific effects of PEGylated recombinant phenylalanine ammonia lyase dosing regimens have been reported in the *Pah^enu2^* mouse model (Sarkissian et al. 2008). Together, these two *Pah^enu2^* murine studies suggest that disease course and management may differ for male and female patients with PKU and deserves further investigation.

This murine study also highlights the role of high dietary intake of Phe on renal dysfunction in PKU. We demonstrated that high dietary Phe intake in *Pah^−/−^* mice fed the casein diet induced polyuria and increased urinary excretion of renal net acid, ammonium, and calcium in addition to mild medullary tubular dilatation, which suggests renal dysfunction. This finding is not surprising given that high intake of Phe in the context of *Pah* deficiency increases the synthesis of several weak organic acids, including phenyllactate and phenylacetate, which must be excreted to maintain acid–base balance (Lemann et al. 2003; Hennermann et al. 2013; Schuck et al. 2015). Therefore, a potential disease mechanism contributing to renal dysfunction in PKU may be partially due to the increased metabolic acid load with increased synthesis of organic acids from altered Phe metabolism with *Pah* deficiency. However, in contrast to the greater renal mass in mice fed the AA and BAA diets, renal mass was significantly lower in mice fed the low‐acid, high‐Phe casein diet compared to the high‐acid, low‐Phe AA diet. This suggests that the casein diet, despite inducing polyuria, provides a lower renal workload relative to the high‐acid AA diet.

Our murine study suggests that chronic ingestion of elemental AAs may be a greater contributor to renal dysfunction in PKU than dietary acid load. This conclusion is supported by the increased renal mass, increased urinary volume, and medullary tubular dilation in male *Pah^−/−^* mice fed the low‐acid BAA diet as compared to the low‐acid GMP diet; this may be due to diuresis for nitrogen detoxification. However, measures of renal function, such as proteinuria and aminoaciduria, to complement our findings of altered renal microarchitecture and confirm proximal tubular dysfunction, were not assessed. Furthermore, the medullary tubular dilation and cortical vacuolation in the proximal tubules were mild to moderate in severity and sex‐specific. It is important to note that the mice in this study were the equivalent of young adults (5–6 months of age); therefore, renal dysfunction in *Pah^−/−^* mice is expected to become more severe with aging (Hennermann et al. 2013).

Consistent with the human studies that show increased rates of osteoporosis in individuals with PKU compared to controls, the *Pah^enu2^* mouse model has demonstrated reduced femoral strength and L_4_ vertebral trabecular mass (bone volume/total volume) partially due to impaired osteoblast proliferation and mineralization (Solverson et al. 2012; Dobrowolski et al. 2018). Similarly, we found reduced femoral maximum load and mineral content in male and female *Pah^−/−^* mice compared to WT littermates with greater decreases in female than male mice. Interestingly, this finding contradicts our human cross‐sectional study that reported lower total body BMD in male versus female subjects with PKU (Stroup et al. 2018).

We hypothesized that ingestion of a high dietary acid load derived from elemental AAs could contribute to reduced bone mass in PKU due to defects in mineralization or altered collagen metabolism (Jehle et al. 2006; Moseley et al. 2012; Solverson et al. 2012; Jehle et al. 2013; Dawson‐Hughes et al. 2015; Dobrowolski et al. 2018). This hypothesis was reasonable given the improvements in BMD and fracture via neutralization of dietary acid load and optimal lysyl oxidase activity, essential for collagen crosslinking, in alkaline in vitro study conditions (Han and Tanzer, 1979; Borel et al. 2001). Interestingly, buffering the AA diet did not improve femoral mineral content or maximum load in female *Pah^−/−^* mice and exacerbated these femoral parameters in male *Pah^−/−^* mice. Additionally, posttranslational modifications in collagen assessed in the femur, tendon, and tail were similar in female WT and *Pah^−/−^* mice, which suggests that suboptimal bone outcomes in *Pah* deficiency are not due to altered collagen metabolism.

In summary, we demonstrated that buffering the AA diet did not improve renal and bone outcomes in male and female *Pah^−/−^* mice. Moreover, renal dysfunction and suboptimal femoral outcomes in *Pah^−/−^* mice were sex‐specific. Although the etiology of renal dysfunction and osteoporosis in PKU remains incompletely understood, this study is the first to report the sex‐specific renal phenotype in the *Pah^enu2^* mouse model and to provide evidence to suggest that *Pah* deficiency does not alter collagen metabolism. Our investigation of the relative contributions of dietary protein source, Phe intake, and acid load suggests that ingestion of elemental AAs is a major contributor to renal dysfunction in PKU. Mechanistic studies investigating the etiology of renal dysfunction and osteoporosis in PKU are needed in order to optimize treatment strategies in PKU.

## Conflict of Interest

Denise M. Ney is co‐inventor on U.S. Patent 8,605,168 B2, “Glycomacropeptide Medical Foods for Nutritional Management of Phenylketonuria and other Metabolic Disorders,” which is held by the Wisconsin Alumni Research Foundation and licensed to Cambrooke Therapeutics, LLC.
